# Task-oriented arm training for stroke patients based on remote handling technology concepts: A feasibility study

**DOI:** 10.3233/THC-220465

**Published:** 2023-09-15

**Authors:** Jule Elmanowski, Melanie Kleynen, Richard P.J. Geers, Gustavo Rovelo-Ruiz, Eva Geurts, Karin Coninx, Jeanine A. Verbunt, Henk A.M. Seelen

**Affiliations:** a Department of Rehabilitation Medicine, Care and Public Health Research Institute, Maastricht University, Maastricht, The Netherlands; b Adelante Centre of Expertise in Rehabilitation and Audiology, Hoensbroek, The Netherlands; c Adelante Rehabilitation Centre, Hoensbroek, The Netherlands; d Research Centre for Nutrition, Lifestyle and Exercise, Faculty of Health, Zuyd University of Applied Sciences, Heerlen, The Netherlands; e Expertise Centre for Digital Media, Hasselt University – tUL – Flanders Make, Diepenbeek, Belgium; f HCI and eHealth, Faculty of Sciences, Hasselt University, Diepenbeek, Belgium

**Keywords:** ReHab-TOAT, technology-assisted, arm-hand skill performance, rehabilitation, stroke

## Abstract

**BACKGROUND:**

Improving arm-hand skill performance is a major therapeutic target in stroke rehabilitation. Arm-hand rehabilitation may be enriched in content and variation by using technology-assisted training. Especially for people with a severely affected arm, technology-assisted training offers more challenging training possibilities.

**OBJECTIVE:**

The aim of this study was to explore the feasibility of ReHab-TOAT, a “Remote Handling Based Task-Oriented Arm Training” approach featuring enriched haptic feedback aimed at improving daily activities and participation.

**METHODS:**

Five subacute or chronic stroke patients suffering moderate to severe arm-hand impairments and five rehabilitation therapists participated. All participants received 2 ReHab-TOAT sessions. Outcome measure was a bespoke feasibility questionnaire on user experiences and satisfaction regarding ‘*motivation*’, ‘*individualization of training*’, ‘*potential training effects*’, and ‘*implementation in rehabilitation*’ of patients and therapists.

**RESULTS:**

Both patients and therapists experienced ReHab-TOAT as being feasible. They found ReHab-TOAT very motivating and challenging. All patients perceived an added value of ReHab-TOAT and would continue the training. Small improvements regarding exercise variability were suggested.

**CONCLUSION:**

ReHab-TOAT seems to be a feasible and very promising training approach for arm-hand rehabilitation of stroke patients with a moderately or severely affected arm. Further research is necessary to investigate potential training effects of ReHab-TOAT.

## 1. Introduction

Between 33 and 66% of stroke survivors in the subacute and chronic phase after stroke have to deal with a moderately or severely affected, hemiparetic arm-hand [1]. In addition to impairments at the International Classification of Functioning, Disability and Health (ICF) [2] function level, like spasticity and pain [3, 4], stroke patients may be severely restricted in their performance of daily activities (ICF activity and participation level) [5, 6]. This limited use of the affected arm in daily life leads to larger caregiver dependence and a reduced quality of life [7]. Especially stroke patients with a moderately to severely affected arm-hand (Utrechtse Arm/hand Test (UAT) [8] score of 1–3) are limited not only in their daily life activities, but also in their training possibilities, the latter further hampering improvements and causing persistence of disabilities. Improving arm-hand skill performance (AHSP) is a major therapeutic target in stroke rehabilitation. 

It has been shown that task-oriented training, i.e. repetitive training of meaningful activities in a functional context, induces changes in the cerebral cortex, supporting motor recovery based on brain plasticity [10, 11] and making learned strategies available for future behaviour [12]. However, given the limited guided treatment time and the limited financial resources for rehabilitation, providing task-oriented training is challenging, because a high treatment frequency and training intensity are necessary to maximize effects [13]. Providing a high training intensity and challenging training environments is even more difficult in patients with a severely affected arm-hand, due to their limited movement possibilities. In order to address this challenge, new technologies are being developed to assist the training of these patients.

A number of systematic reviews about the effectiveness of robot-assisted therapy for upper extremities have been published in the last decade [16, 17]. Although the effect of technology-assisted training on motor recovery in stroke seems promising [18, 19], effect sizes and implementation rates are still low [16, 17]. Also, many of the developed technology-assisted training regimes focus on patients with only minor impairments in their arm-hand function (UAT score 4–7) and especially on patients in the (sub-)acute phase after stroke [20]. However, despite getting less attention in research, it has been shown that significant improvements at function and activity level following technology-assisted treatment are possible even in the chronic stage after stroke [21, 22]. Furthermore, evidence-based, technology-assisted training programs for stroke patients with an UAT score of 1 or 2 are lacking.

Given the afore mentioned, we developed a new task-oriented arm training approach using a so-called ‘remote handling concept’ device featuring haptic feedback, to manipulate proprioception, i.e. the sense of movement, in patients with a severely affected arm-hand (UAT 1–3). This approach is called “Remote Handling concept based, Task-Oriented Arm Training” (acronym: ReHab-TOAT). The intervention consists of task-oriented training, incorporating the principles of motor learning, and is based on the training approach of two previously described and evaluated training concepts, i.e. “CARAS” [23] and “TOAT” [22]. The ReHab-TOAT approach uses small assistive/resistive forces (i.e. haptic feedback), currently mechanically provided by the ‘remote handling concept’ device called Dexter${}^{\text{TM}}$ (Veolia Nuclear Solutions, Didcot, UK). The corresponding software was developed to control the provision of the assistive/resistive forces to the arm of the patient in 3D space, thereby systematically manipulating proprioception, during the performance of different functional movements related to daily activities. By providing haptic feedback, the sensory information the patient receives on his motor performance is enriched, which may improve motor control, motor learning and (long-term) retention, as well as AHSP, resulting in a more comfortable performance of activities of daily living. A small number of studies already exist, which examine the effects of technology-assisted, sensory retraining protocols on upper limb impairment and upper limb function post-stroke. However, no results on the feasibility of these training protocols have been reported to date [24, 25, 26, 27]. A first step in the evaluation of the developed ReHab-TOAT approach has been to explore its feasibility for patients and therapists within the current arm-hand rehabilitation of stroke patients. Satisfaction, especially regarding the motivational and individualization aspects of ReHab-TOAT, the intention to continue the use, and the fit within current rehabilitation regimes, are important aspects of acceptability and are necessary for successful implementation [28]. Another important aspect of feasibility is practicality regarding the extent to which the training approach can be carried out. Furthermore, systematically gauging the perceived potential positive and/or negative consequences for participants is important in this feasibility study, because it may lead to further improvement of the training approach and may provide insights for the selection of adequate measures to be used in a later study on effectiveness [28].

Therefore, the aim of the present study was to assess the feasibility of the ReHab-TOAT approach featuring enriched haptic feedback aimed at improving activities of daily living and participation in chronic and in subacute stroke patients with either a moderately or severely affected arm-hand. This was done from both the patients’ perspective and the therapists’ perspective. Special emphasis was put on motivation, individualization of training, training effects, and implementation in a rehabilitation context.

## 2. Methods

This feasibility study was approved by the Medical Ethics Committee of Maxima Medisch Centrum in Veldhoven, the Netherlands (study code: NL70014.015.19). The study was also registered in the ISRCTN registry (ISRCTN50551089).

### 2.1 Participants

Five patients in either the subacute or chronic phase after a stroke participated. Patients were identified from the database of the department of brain injury rehabilitation of Adelante rehabilitation centre in Hoensbroek, the Netherlands. They all met the following inclusion criteria: unilateral stroke; post-stroke time between 6 and 12 weeks (subacute) or post-stroke time larger than 12 months (chronic); arm-hand motor impairment, i.e. an Utrechtse Arm/hand Test (UAT) score of 1–3 [8]; age > 18 years; sufficient cognitive abilities to understand the questions posed in the questionnaires and the measurement instructions. Exclusion criteria were: spasticity in the affected upper limb, i.e. a modified Ashworth scale (MAS) score > 1+ [29]; non-stroke-related comorbidity that may interfere with arm-hand function.

Also, five therapists participated in the study. They all met the following inclusion criteria: working at a specialized rehabilitation centre, i.e. Adelante Zorggroep, Hoensbroek in the Netherlands; holding a degree in physiotherapy or occupational therapy; having at least 6 years of experience in the treatment of patients with central nervous system deficits.

### 2.2 ReHab-TOAT

**Figure 1.**
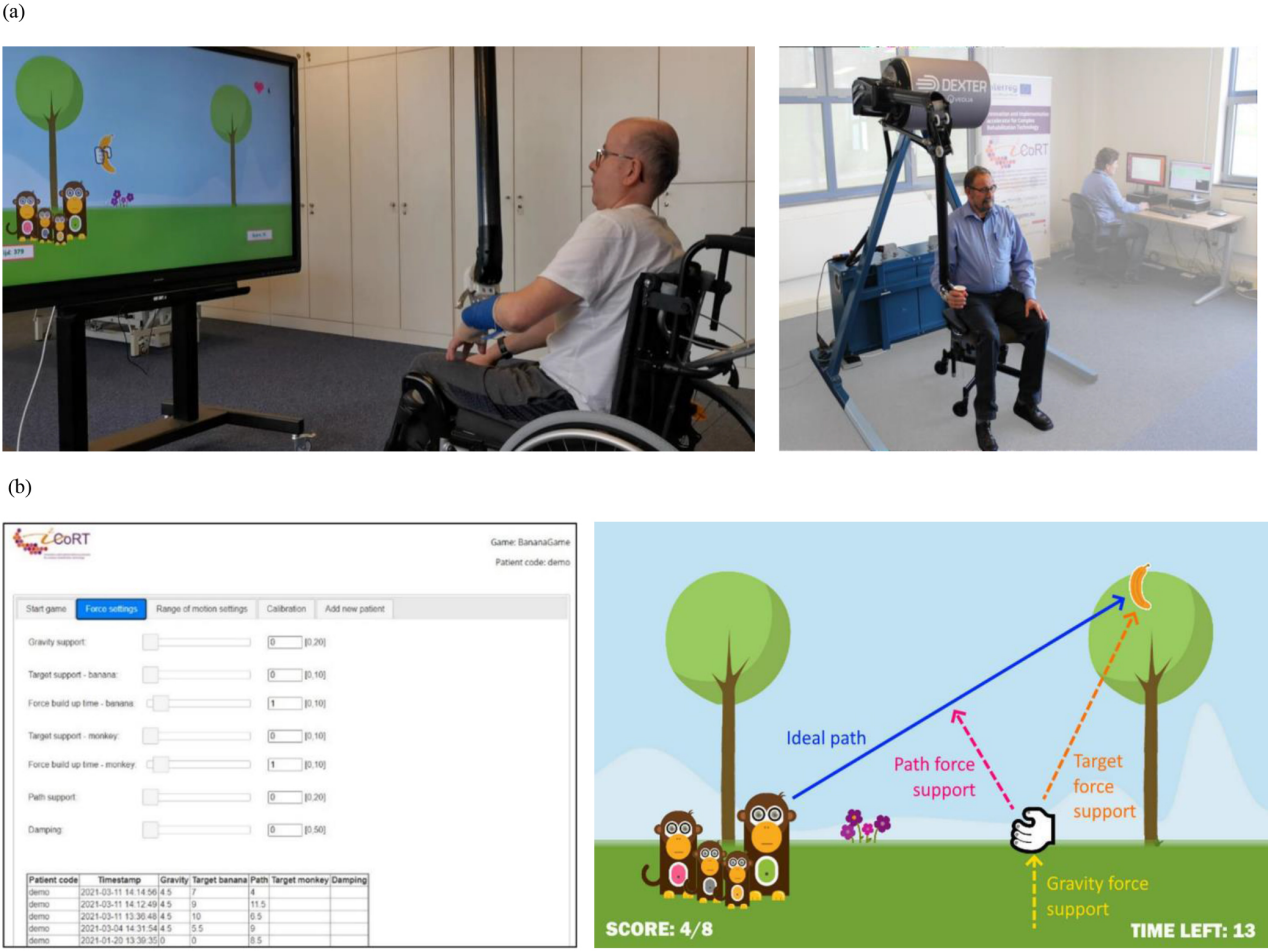
(1a) Dexter${}^{\text{TM}}$ training set-up and (1b) therapist interface & example of force directions. (1a) The left picture shows the training set-up with a patient. The right picture shows the Dexter${}^{\text{TM}}$ device itself, and the technical expert with the necessary computers in the background. (1b) The left picture shows the interface through which the therapist can set and change training settings of the Dexter${}^{\text{TM}}$ device. The right picture illustrates the different forces that can be defined in a game and can be personalized for each patient.

ReHab-TOAT is a task-oriented arm training approach for stroke patients with a moderately to severely affected arm-hand (UAT score 1, 2, or 3) [8]. One of ReHab-TOAT’s unique features is that the therapists can provide (and instantly change) enriched haptic feedback, generated by the ‘remote handling concept’ device (see Fig. 1), on an “as-needed” basis, related to the (daily) skills that are being trained on.

**Figure 2.**
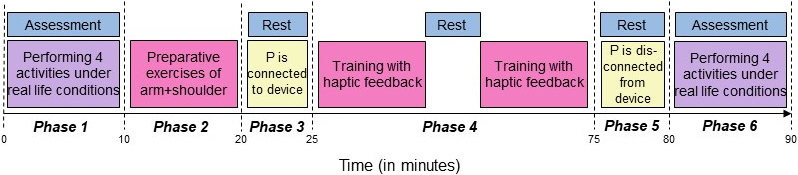
ReHab-TOAT training session timing.

The general content and time planning of each training session of the ReHab-TOAT approach is depicted in Fig. 2. Each session is subdivided into six phases. Phase 1 is similar to phase 6 and contains the performance of daily arm-hand activities the patient wants to improve on. These phases are also used to assess patient’s arm-hand status (before and after training). Phase 2 contains several preparation procedures. Phases 3 and 5 are also similar to each other and feature a resting period for the patient while a therapist connects or disconnects the patient to and from the arm orthosis attached to the Dexter${}^{\text{TM}}$ device (see also Fig. 1). Phase 4 is the main training phase, and also several resting periods.

A comprehensive description of the ReHab-TOAT approach, including training session timing, training content & training build-up, and the potential for generalization of training effects, are presented in Appendix 1, 2 and 3 respectively.

ReHab-TOAT was originally developed to be provided in three sessions per week, each session lasting 1.5 hours, over a training period of four weeks, based on previous research [9, 23]. However, for the present feasibility study each participant received one or two training sessions of ReHab-TOAT in order to a) evaluate the feasibility of a single training session, and b) to be able to fine-tune the training protocol.

### 2.3 Outcome measures 

Outcome measures were user satisfaction and experiences from both patients and therapists regarding different aspects of feasibility [28]. The outcome measures were categorized in ‘motivation’, ‘individualization of training’, ‘possible training effects’ and ‘implementation in the rehabilitation context’, i.e. the patient’s and therapist’s opinion regarding ReHab-TOAT as possible treatment within arm-hand rehabilitation of stroke patients. As ReHab-TOAT not only consists of technology, but also (novel) clinical training approaches and intricate interactions between these two fields, the afore mentioned outcome variables were gauged using a bespoke feasibility questionnaire rather than using existing questionnaires covering only one of either fields. This questionnaire was developed in a combined effort of experts in clinical rehabilitation, medical technology, methodology & psychometrics, health sciences and human-computer interaction. This questionnaire consisted of two parts. The first part contained 11 questions on motivation, training intensity & difficulty, individualization, and added value of the training, each rated on a 7-point Likert scale (1 point = I totally disagree, 7 points = I totally agree). The second part of the questionnaire contained six open questions on individual experience and satisfaction of training content, training intensity, possible training effects, added value for arm-hand rehabilitation of stroke patients, and possible improvements for further optimization of ReHab-TOAT. The questionnaires were filled out by the patients themselves after they had received the last training session of ReHab-TOAT. If the patient was unable to fill out the questionnaire, due to problems with writing, the therapist present filled in the answers verbatim, only using the patient’s own words.

### 2.4 Data analysis

The data of the quantitative part of the questionnaires from therapists and patients are reported descriptively. The answers to the open-ended questions were analysed using directed content analysis [30]. The initial coding scheme was based on the themes included in the questionnaire. This scheme was extended through analysis of the data. The main themes were described, quantified using frequency counts, and illustrated with quotes.

## 3. Results

### 3.1 Participants

The characteristics of the 5 stroke patients and the 5 therapists who participated in this study are shown in Table 1a and 1b.

**Table 1a:** Participant demographics of patients

Patient	Age (yrs)	Sex	Side of stroke	Post-stroke time (months)	UAT score	Spasticity level (MAS)
P1	63	M	Left	119	3	1 (arm, wrist, fingers)
P2	54	M	Right	19	2	1 (arm, wrist, fingers)
P3	67	F	Right	2	1	1 (arm)
P4	58	M	Right	24	3	1 (arm, wrist)
P5	63	M	Right	2	3	0

${}^{*}$F = Female; M = Male; P = Patient; UAT = Utrechtse Arm/hand Test; MAS = Modified Ashworth Scale.

**Table 1b:** Participant demographics of therapists

Therapist	Age (yrs)	Sex	Profession & degree	Specialism	Working experience (years)	Experience using technology in rehabilitation (yrs)
T1	29	F	OT, BSc	Neurological disorders	7	3
T2	43	M	OT, MSc, researcher	Arm-hand rehabilitation	20	8
T3	30	F	PT, BSc, teacher	Neurological disorders, chronic pain	6	4
T4	31	F	OT, BSc	Neurological disorders	7	1
T5	44	M	PT, MSc	Neurological disorders	21	8

${}^{*}$F = Female; M = Male; OT = Occupational therapist; PT = Physical therapist; BSc = Bachelor of Science; MSc = Master of Science.

### 3.2 Feasibility questionnaire

The individual results of the quantitative part of the feasibility questionnaire of the patients are presented in Table 2.

**Table 2:** Results of patients regarding feasibility questionnaire

Question	P1	P2	P3	P4	P5
1. This arm-hand training was fun to do.	7	7	7	7	7
2. For me, it was clear what I needed to do during the exercises/games.	7	6	7	7	7
3. I was motivated by the exercises/games to do my best.	6	6	7	7	5
4. I found the exercises/games I had to do too difficult.	1	1	1	3	1
5. I liked the duration of the training.	4	6	6	7	4
6. The training was too hard to me.	1	2	2	4	1
7. I found this training useful to do.	6	7	7	7	7
8. I found this arm hand workout boring to do.	6	1	1	1	1
9. I found this training fatiguing or exhausting.	1	4	3	4	4
10. I think this training is important for improving my arm-hand skill performance.	5	6	6	7	7
11. I would like to do this training again because I think that I would benefit from it.	6	7	4	7	7

Likert scale: 1 point = I totally disagree; 4 points = Neutral; 7 points = I totally agree. P = Patient.

All five patients were very positive about ReHab-TOAT. They all rated the enjoyment of ReHab-TOAT with the maximum score of 7 points. Only one patient found ReHab-TOAT “*a bit boring*” and “*not exhausting*”. In general, the patients were positive about the motivational aspect of ReHab-TOAT. They used terms like “*nice*” (P⁢2), “*good for the arm*” (P⁢4) and “*challenging*” (P⁢2). For one patient, however, the games were “*not challenging enough*” (P⁢1). Three out of the five patients were most content about the training effect they experienced immediately after training, and which seemed to be retained at home: “*This training has an added value for stroke patients like me. Due to the training, activities I performed afterwards in my own home situation were much easier to do*.” (P⁢1). The other two patients were most satisfied with the clear training instructions of ReHab-TOAT and the clarity of the training objective: “*Clear what I have to do and what is the goal of the training*” (P⁢4). Different aspects of the individualized training were experienced as “*easy*” (P⁢4) by the patients: for example “*understanding the exercises*” (P⁢2), “*support by the robot*” (P⁢3), and “*the whole training*” (P⁢1). Three patients found the training to be physically demanding, while one patient found it to be cognitively demanding: “*Execution requires concentration, anything that interfered with this had an impact on my performance*.” (P⁢5).

In addition, patients made suggestions on possible improvements for further development of the training approach to optimize implementation of ReHab-TOAT in a rehabilitation setting. Four of them suggested to include more variations, for example by adding increasing levels of difficulty and making more use of the whole range of motion (ROM) of the movement possibilities of the patient during one exercise, “*I would like to use the whole playing field during the games to make more movements during one exercise*.” (P⁢1). Two patients suggested adding other movements to the games, “*The game in which we had to feed bananas to the monkeys can be expanded by adding grasping and releasing the banana with our fingers*.” (P⁢4). One patient suggested to adjust the duration of the training for subacute patients, because he was “*afraid that it might be too long for other patients, but for me it was perfect*” (P1). Another patient stated the importance of the presence of a therapist during a training session “*to make sure that there is therapeutic added value of the training*” (P⁢5). Two patients would like to continue the training as it is, “*Sure, because I think that this training has a positive effect on my arm-hand function*” (P⁢5), while three would like to continue the training with small adaptations regarding the variation of the training, “*Yes, gladly with an increase in challenges*.” (P⁢2).

The individual results of the quantitative part of the feasibility questionnaire from the therapists can be found in Table 3.

**Table 3:** Results of therapists regarding feasibility questionnaire

Question	T1	T2	T3	T4	T5
1. This arm-hand training was fun to do.	6	5	7	5	5
2. For me, it was clear what I needed to do during the exercises/games.	4	6	6	7	7
3. I think the exercises/games I had to do are also motivating for patients with a stroke.	6	5	6	4	5
4. I think the exercises/games I had to do are too complex for patients with a stroke.	3	3	2	1	2
5. I think the duration of training is good for patients with a stroke.	5	4	6	7	6
6. I think the exercises/games I had to do are appropriate in intensity for the level of patients with	5	5	6	7	5
a stroke.					
7. I think this training is useful to do for patients with a stroke.	4	5	7	6	5
8. I found this arm hand workout boring to do.	2	2	1	3	2
9. I think this training is too exhausting for patients with a stroke.	5	4	2	1	3
10. I think this training is important for improving arm-hand skill performance in patients with a	4	5	6	7	5
stroke.					
11. I think this training has an added value to the arm-hand rehabilitation of patients with a stroke.	5	6	7	7	6

Likert scale: 1 point: I totally disagree; 4 points: Neutral; 7 points: I totally agree. T = Therapist.

Regarding the open questions, all five therapists stated that they liked ReHab-TOAT. Three of them especially liked the motivating way of training, “*Challenging and inviting to push your own limits*” (T⁢5). Three therapists most of all liked the high number of adaptations and variations which can be made by the therapist: “*Many possibilities to do arm-hand training also in patients with little arm-hand function*” (T⁢1). The therapists made suggestions on possible improvements regarding different aspects of the training approach to optimize implementation of ReHab-TOAT in a rehabilitation setting. All therapists stated that they would like to have even more variation during the training. For example, all five therapists stated that they would like to see more exercises (or a higher ROM within the exercises) regarding the hand and fingers: “*Other movements like dorsal and palmar flexion of the hand can be added to extend the ROM even wider*.” (T⁢4). Three therapists suggested to add more games and exercises in general. Four therapists would like to include more evaluation and feedback moments during and at the end of the exercises: “*Let the monkey smile if the patient was successful in giving the banana to the monkey*.” (T⁢3). Two therapists suggested to include a countdown before the exercise starts, and both suggested to show to the patient the duration of the exercise as well as the pauses. Two therapists suggested to improve the software by adding “*the possibility to generate resistance during the exercises, because resistance against the performed movement can support patients with tremors to better coordinate their movement*” (T⁢5). Two other therapists suggested to improve the hardware, first by minimizing the size of the robotic device. One therapist stated that “*the whole robotic device should become smaller*” (T⁢3), while another therapist stated that only the gimbal which connects the arm of the patient to the robotic device should become smaller (T4). Two therapists would use the training approach in patients after stroke: “*Yes, the device seems to be easy to operate and many settings are possible*” (T⁢4), while three therapists would use it under special conditions: “*Sure, take into account if someone has special needs because of a painful shoulder*” (T⁢5).

## 4. Discussion

The aim of this study was to assess the feasibility of the use of the ReHab-TOAT approach in chronic and in subacute stroke patients with either a moderately or severely affected arm-hand, from both the patients’ perspective and the therapists’ perspective regarding motivation, individualization of training, training effects, and implementation in a rehabilitation context. As far as we know this is one of the first studies describing the development and exploring the feasibility of a technology-assisted training approach for patients with a severely affected arm-hand in the chronic stage after stroke. The results of this feasibility study are very promising. In general, ReHab-TOAT was experienced by both the patients and the therapists as being feasible. They experienced the training as very motivating and challenging. Only small improvements regarding the exercise variability of ReHab-TOAT were suggested. All patients perceived an added value of ReHab-TOAT in their treatment and would continue the training if the opportunity for it would arise. Furthermore, all therapists would like to use ReHab-TOAT as a part of treatment during the current arm-hand rehabilitation of stroke patients.

### 4.1 Motivation 

Nearly all patients and therapists found ReHab-TOAT fun, motivating and useful. From both a therapist’s and a patient’s point of view, the high number of possibilities regarding settings and graphics, i.e. the images and figures generated by a computer in the digital environment of the ReHab-TOAT system, led to challenging training sessions with variations, also for patients with a severely affected arm-hand. This is in line with previous studies [31, 32]. Research has shown that, in a non-game environment, the application of game elements to support user engagement and fun [33] improves the motivation of patients during training, especially during repetitive movements, and facilitates patients’ rehabilitation at ICF activity level [34, 35]. However, the results of our feasibility study indicate that even more variations are desirable for a successful implementation in a rehabilitation context.

### 4.2 Individualisation of training

Our feasibility study shows that individualization of therapy is possible within ReHab-TOAT, which is consistent with previous research indicating that individualization of treatment is necessary for stroke patients to achieve optimal recovery [36]. Patients and therapists valued the haptic feedback generated by the robotic device, because it creates the possibility to train meaningful movements in a motivating and useful way, even in patients with a low arm-hand function or patients with cognitive impairments. They also appreciated the possibility to be completely free in choosing the settings for each training session and being able to instantly adapt the settings during the exercises, based on the individual needs of the patient.

### 4.3 Potential training effect

Where many other technology-assisted arm-hand training approaches focus on ICF function level [17], ReHab-TOAT mainly focuses on training at ICF activity level, supported by aspects of intrinsic motivation, self-efficacy, and behavioural change. In line with previous research [37, 38, 39], our feasibility study has shown that patients and therapists appreciate the versatility and all-in-one training approach of ReHab-TOAT, which may lead to clinically relevant improvement of both self-perceived and actual skill performance in daily life and quality of life. Also, restoring the patients’ trust in their arm abilities may lead to even higher improvements on all levels of the ICF model. Training meaningful activities may increase the chance that the patient applies these newly acquired activities in his/her home environment, thus augmenting repetition of training and stimulating retainment of skills.

Whether the haptic feedback provided in ReHab-TOAT indeed leads to improvements in coordination of movements and thereby also to improved AHSP, and whether these improvements are larger than the effect sizes reported in papers on currently used technology-assisted training, has yet to be demonstrated by an ensuing Randomized Clinical Trial (RCT). However, one added value of using technology-assisted force feedback in ReHab-TOAT is that the training is made accessible to a broader group of patients. It creates the possibility to train meaningful movements even in patients with a severely affected arm-hand function or in patients with cognitive impairments.

### 4.4 Implementation in rehabilitation context 

In our study, both patients and therapists agreed that ReHab-TOAT has an added value for arm-hand rehabilitation of stroke patients, because of a) force feedback making therapy possible for a broader range of patients; b) the self-perceived training effects in daily life; c) the fun element; d) the challenging and motivating way of training; and e) the incorporation of self-efficacy principles and the stimulation of autonomy of the patient during training. And as therapists and patients, besides other experts, were involved in the development process of ReHab-TOAT right from the start of the project conception, their ideas and thoughts on the use of rehabilitation technology, and more specifically the use of the Dexter${}^{\text{TM}}$ device, have been incorporated in the project and the design of the treatment protocols. This involvement of patients and therapists at an early stage in the development process is an essential element for successful implementation in future. Technology-assisted arm training may not only augment the amount and the duration of supervised and unsupervised training, but may also augment content variety and task specificity of training. It may thereby provide optimal conditions for challenging the patient’s brain plasticity regarding sensorimotor (re-)learning, yet, at the same time, keep the workload for (para-)medical staff and treatment costs manageable [14, 15].

Also, in order to further optimise ReHab-TOAT, during the execution of the project several of the participants suggested to add more games and exercises, which enhances practice variability and training motivation. This, in turn, may increase generalization and transfer of training effects towards novel situations and other arm-hand skills [40]. Therapists suggested to add fixed feedback and evaluation moments during ReHab-TOAT, which is in line with clinical expertise of the authors and with previous research indicating that feedback and evaluation of training result in further improvement and generalizability of training in other contexts [10, 41]. The other suggestions given by therapists, like a smaller size of the robotic device to fit in different training locations and practicing in groups, indicate that, at this stage, the therapists are already thinking about possible future applications in rehabilitation and the possibilities to embed the ReHab-TOAT concept in regular clinical practice. All these suggestions by therapists and patients and the end-user involvement in the project may fine-tune solid actual implementation of ReHab-TOAT in daily rehabilitation practice.

### 4.5 Strength and limitations 

The purpose of the present study was to assess the feasibility of ReHab-TOAT. Feasibility can be sub-classified in several key areas of interest, as described by Bowen and co-workers [26]. We focused especially on the items: ‘Acceptability’, i.e. ‘satisfaction’ regarding the motivational and individualization aspects of ReHab-TOAT, ‘intent to continue the use’, and ‘fit within the current treatment concepts’; ‘Practicality’, i.e. the extent to which the training approach can be carried out; and ‘Demand’/‘Integration’, i.e. the perceived fit within the current rehabilitation processes, and the perceived sustainability. To accommodate this, small groups of participants do suffice.

The strength of this study is that ReHab-TOAT was tested by a defined group of therapists (with various backgrounds as to the level of experience working with central nervous deficit patients as well as level of experience in using technology) and patients (stroke patients in chronic and subacute stage after stroke with a severely or moderately impaired arm-hand function and from different ages), in order to obtain a comprehensive set of information on the feasibility. This variety in participants was of added value, as it provided us with relevant and broad information on potential future end-users. ReHab-TOAT was tested under realistic circumstances, i.e. in regular rehabilitation conditions, in which it will also be used in the future. Furthermore, ReHab-TOAT has been developed for a wide group of patients, and contains all necessary and evidence-based training principles of current arm-hand rehabilitation regimes.

During this study participants gave insights into their self-perceived performance and self-perceived training effects after only two sessions of ReHab-TOAT. Consequently, potential experienced effects that might occur after a longer use of the approach were not investigated. The same applies to aspects of the feasibility which might change during a more long-term use of the approach, especially regarding the motivation of patients. Furthermore, as none of the existing questionnaires gauging (health care) technology was specific enough for our queries regarding ReHab-TOAT, two bespoke questionnaires were constructed, based on expert opinion from different domain experts (see Appendix 4 and 5). These questionnaires were not validated against any existing criterion or existing questionnaires.

Our study did not focus on feasibility items like ‘Adaptation’, ‘Expansion’ or ‘Limited efficacy’. The latter would have necessitated a far larger number of participants in larger (and homogeneous) strata or subgroups, and a different methodological study set-up contrasting conditions and/or subgroups. No objective measures on any potential physical improvements and training effects were gauged.

## 5. Conclusion 

ReHab-TOAT seems to be a feasible training approach for arm rehabilitation of stroke patients with a moderately or severely affected arm. Both patients and therapists identified several benefits of ReHab-TOAT, e.g. the haptic feedback, increased motivation, enhancing self-efficacy and autonomy of the patient during the rehabilitation process, the possibility of individualizing the training (based on the patients’ needs), and the possible training effects at different levels of the ICF model. All participants wanted to continue the use of ReHab-TOAT, because they saw the high potential of gaining improvements, not only at arm-hand function level, but particularly at activity and participation level. Further research is necessary to investigate any potential training effect of ReHab-TOAT on patients’ performance at the different levels of the ICF model.

## Funding 

This study, as part of the i2-CoRT project (www.i2-CoRT.eu), has been co-funded by the Interreg V-A Euregio Meuse-Rhine (EMR) programme under Grant EMR1. The Interreg EMR program has invested almost EUR 100 million in the development of the Interreg-region until 2020. With the investment of EU funds in Interreg projects, the European Union directly invests in the economic development, innovation, territorial development, social inclusion and education of this region.

## Data availability statement 

Coded data will be made available to the scientific community upon reasonable request.

## Supplementary data

The supplementary files are available to download from http://dx.doi.org/10.3233/THC-220465.

## Supplementary Material

Appendices
