# Supplementary material for: Task-oriented arm training for stroke patients based on remote handling technology concepts: A feasibility study
Source: Technol Health Care. 2023 Sep 15;31(5):1593–605. doi: 10.3233/THC-220465 (PMC10578292; doi:10.3233/THC-220465)
Supplement: Appendices [file thc-31-thc220465-s001.docx]

# **Appendices**

## ***Appendix 1: ReHab-TOAT training session***

### Training session phase 1

One of the first actions during each week of the four weeks ReHab-TOAT approach is to identify four daily life arm-hand tasks/skills the patient wants to improve on during that week, consisting of one static task (i.e. stabilizing vegetable while cutting them), one dynamic task (i.e. washing the non-affected arm) and two self-selected tasks. At the start of each ReHab-TOAT training session (phase 1), the patient undergoes a baseline assessment containing the following steps:

- Firstly, a physical therapist or occupational therapist performs a skill analysis for each of the four tasks selected, while the patient performs those tasks under real-life conditions. This skill analysis contains several steps to: a) quantify the patient’s functional abilities; b) check the strategies used to accomplish functional skills and c) consider which underlying sensory, motor or cognitive factors constrain functional movement, and which factors are the most important to train on. Also, the patient is asked “What would you, as a patient, like to improve on during the upcoming training session?”. (S)he is also asked to rate his/her perceived performance on each of the tasks selected earlier, answering the following question: “How well do I, as a patient, think I already can perform this task?”. Here a visual analogue scale (VAS) is used.
- Secondly, a suitable motor learning approach and appropriate practice conditions will be recorded in a personal treatment plan that is then discussed with the patient. This treatment plan can be adapted as training progresses over time. Content and initial parameter settings for the technology-assisted part of the training are then defined and converted into different exercises and games which will be offered to the patient in training session phase 4. The selection of the actual exercises and games to be used for a specific patient during ReHab-TOAT training in phase 4 (see below) is also determined by the motor deficits experienced during the performance of activities of daily living and the meaningful goals identified by the patient. For example, if a patient wants to improve on stabilizing vegetables while cutting with the non-affected hand, the therapist often chooses for a reaching exercise combining protraction and retraction movements of the shoulder/arm. If the therapist observes that the patient has difficulties with moving his arm, for example when the patient uses compensatory movements like elevation of the shoulder, the therapist may choose to use anti-gravity support for the arm. Based on the amount of compensation observed, the estimated weight of the arm of the patient, and the experience of the therapist with the forces of the Dexter^TM^ device, (s)he may estimate the amount of anti-gravity support needed. Based on the coordination and control of the observed movement, the therapist may also choose to have the Dexter^TM^ system apply other forces, like a force aiding the patient’s arm in the direction of the movement, or applying forces resisting the direction of a movement, to stimulate a controlled movement sequence of the affected arm. Furthermore, the therapist may set the duration and number of repetitions of the game, based on knowledge of what is necessary to perform the activity of daily living and what is possible for the patient at a certain moment in time / during training. These parameters are used in the first training session and can be adapted instantaneously if needed. Therefore, it is important that the therapist observes the movements of the patient, look out for compensatory and/or uncontrolled movements, and then analyses the reason behind these deficits in order to adapt training parameters adequately.

### Training session phase 2

In training session phase 2 the patient, guided by the therapist, performs a small number of preparative exercises for the arm and shoulder joints and muscles before the actual training starts. The first part of these exercises is aimed at improving and maintaining an optimal condition of the affected arm and shoulder girdle, i.e. supple and pain free. The second part of these exercises consists of scapula stabilising exercises combined with glenohumeral joint stability exercises.

### Training session phase 3

In the third phase of the training session, the patient has a short resting period of five minutes while (s)he is positioned in front of and connected to the Dexter^TM^ device by a trained physical therapist or a trained occupational therapist. The positioning of both the patient and the chair, relative to the Dexter^TM^ device, is protocolled in order to optimally make use of the 3D movement volume of the device. The patient is seated in front of the device, and the chair is adjusted to fit the patient’s individual anthropometric measures. The patient sits in a comfortable upright sitting position with both feet on the floor, to prevent sliding, and his/her back is well-supported by the backrest, leaving the scapulae free. Armrests are not needed as they can hamper or even block movements. Alternatively, a patient may use his own wheelchair, adapted to his personal needs. Depending on the individual anthropometric size of the patient’s wrist, an appropriately sized arm orthosis is selected and attached to the Dexter^TM^ device, subsequently attached to the patient’s forearm and secured using Velcro straps. Wrist stability is checked for.

### Training session phase 4

Following training session phase 3, the main training part in which the Dexter^TM^ remote handling device is used, starts. It consists of (a maximum of) 50 minutes of training with at least one resting period of ten minutes half way, and several smaller resting periods between exercises. The therapist programs / sets exercises for each patient, based on the individual needs and impairments. The set-up of these practice conditions is closely related to the patient’s functional abilities and training goals, identified during training session phase 1. The tasks/skills, identified in training session phase 1 as being important to the patient, are segmented into sub-tasks (or dominant movement components) that each can be trained on. Different exercises / games are offered to the patient via a screen in front of him/her to train these sub-tasks / movement components using enriched proprioceptive information delivered by the Dexter^TM^ device during (sub)task execution. Setting training parameters, like movement speed, movement assistance/resistance related to force feedback experienced by the patient, additional (visual or auditory) cues, movement direction & extent, (maximal) spatial movement volume, dwell time on specific targets within each game/exercise, number of repetitions, training time etc. (see also appendix 2), is done via a software-driven interface operated by the therapist (see Figure 1b).

During task performance, the patient may use different strategies to explore his/her motor workspace. This exploration involves perceptual cues in order to identify those strategies that are most relevant to the performance of a specific task. Thus, to shift towards optimal strategies involves not only the appropriate motor response, but also finding the most appropriate perceptual cues. During the main training part of ReHab-TOAT, the patient will train meaningful movement components of the previously performed daily life arm-hand tasks/skills, while receiving task-relevant perceptual information/cues in terms of haptic feedback, generated by Dexter^TM^. This perceptual information is provided to the patient *during* the movement, in order to establish an optimal task-relevant learning of perception and action. When the patient experiences improvements in task performance and selects more appropriate strategies towards task accomplishment (observed by a therapist), the degree of haptic feedback provided by Dexter^TM^ will be reduced. The idea is, that the patient, at this point, gradually learns to control his/her own degrees of freedom and learns to control the re-acquired movement skills.

The practical application of the training content is based on: a) Carr and Shepherd’s concept of gradual ‘increase of movement degrees of freedom’ that are to be controlled [42], and b) ‘task segmentation’, i.e. the breakdown of skills in functional movement components that will be practiced, first isolated and later in combinations (‘chaining’) [43, 44]. Examples of task segmentation and movement components are given in Figure 3.

**Figure 3 about here.**

The patient learns to master each individual step prior to learning the entire task. During training, the patient may practice several of these components within the exercises and games. Based on the skill assessments and task requirements, some movement components will be trained more extensively than others. The patient trains in blocks where one movement component or subskill is trained for repetitively for some time (block of trials), after which the patient progresses to the next block of movement component / subskill training. When the patient experiences progression, subskill training is offered in random order conditions or in conditions where all subskills are combined into a complete skill in a different exercise or game, reflecting the whole task or activity. The shift from part-practice to whole practice demands the use of more patterns of movement coordination and demands the use of a variety of motor programs [45]. Ultimately, this may also lead to task generalization towards other untrained tasks (i.e. inter-task transfer of learning) [46] and towards tasks being adequately performed in different circumstances [10].

### Training session phase 5

Following training session phase 4, the patient, again, has a short resting period of five minutes, while (s)he is disconnected from the Dexter^TM^ device by a therapist. Next, the therapist removes the tubular bandage and examines the skin area where the arm orthosis was attached to the forearm to inspect the skin for any possible signs of skin irritations (e.g. redness) due to the patient having worn the orthosis.

### Training session phase 6

The ReHab-TOAT ends with training session phase 6, where the patient has to perform the four activities from the first phase of the training session again. After having finished each activity, the therapist will gauge the patient’s performance once more. The patient will rate his/her perceived performance on the activity on a VAS scale again, only to be used for clinical purposes.

Furthermore, ReHab-TOAT is developed in such a way that it may be used during group training, where each participant works on his/her own goals guided by a therapist. During group training, a therapist can choose the technical part of ReHab-TOAT (phase 4) as one treatment possibility for each patient, while the other patients receive for example mobilization exercises or task-oriented training from previous phases of ReHab-TOAT during this time, and swap afterwards. By working in a group, patients may learn from each other, creating the feeling of social commonality and understanding.

## ***Appendix 2: ReHab-TOAT training parameters and training build-up***

During ReHab-TOAT the patient will train for four weeks, 3 times per week, 1.5 hour per day. As mentioned above, there are at least 3 resting periods of approximately five to ten minutes in between training session phases to avoid overuse and fatigue. The Dexter^TM^ device and the associated (gaming) software offer several opportunities to adapt the ReHab-TOAT treatment content and treatment difficulty, including settings related to, among others:

- (max.) spatial movement volume, i.e. the 3D area in which the patient can move his/her upper limb, which can be adapted before and during the exercises to the patient’s current functional arm status;
- movement direction;
- movement extent / range of motion;
- movement speed;
- movement assistance/resistance related to force and force feedback experienced by the patient during the exercises;
- force build up time at any time during exercise;
- movement damping at any time during exercise;
- arm weight support at any time during exercise;
- dwell time on specific targets within each game/exercise;
- additional visual or auditory cues;
- sequence/order of different exercises;
- number of repetitions;
- number of sets of exercises;
- training time / game duration.

ReHab-TOAT combines principles of training physiology (e.g. patient’s goal-dependent training load), motor learning (e.g. feedback, exercise variability and implicit/explicit learning), and both the Bio-Psycho-Social (BPS) model and the ICF model [10, 47-49]. ReHab-TOAT consists of several levels of difficulty, training extent, and exercise variability, based on different training principles from:

- training physiology, i.e.:
  - training intensity, determined by e.g. the number of sets of exercises / skills trained on, and the number of repetitions [47];
  - density of training content, as determined by the rate between stimuli and resting periods [47];
  - training extent, determined by Range-Of-Motion and movement support [44, 48];
  - training overload, super compensation and specificity [47];
- motor learning, i.e.:
  - feedback [44, 48];
  - implicit and explicit motor learning [48];
  - training content variability;
  - part-practice, i.e. task segmentation, including training of movement components;
  - total skill training (“chaining”);
  - blocked and random practice [50];
  - massed practice [44, 48];
- BPS & ICF model, i.e.:
  - combination of training at the level of function, activity and participation
    [2];
  - stimulating intrinsic motivation by setting and working on meaningful
    rehabilitation goals [39, 51];
  - stimulating self-efficacy by setting and working on own rehabilitation goals
    [37, 52];
  - stimulating behavioural change, i.e. using the affected arm in daily life
    activities by increasing trust in the affected arm’s abilities [53];
  - evaluate improvements in daily life and personal/emotional status as well
    as training progress, and discuss their influence on each other [9, 23];
  - involve caregiver or other important family members in training,
    homework assignments and behavioural change [53];
  - generalization of training effects towards home situation and other
    circumstances [38, 53].

Based on the patient’s individual needs and impairments (personalized, client-centred rehabilitation treatment concept), the therapist chooses and adapts the level of difficulty, training extent and exercise variability during the ReHab-TOAT session(s) in order to obtain an adequate training response, but at the same time preventing (signs of) overuse [47].

## ***Appendix 3: Generalization of training results***

A very important issue in arm-hand rehabilitation is how any ReHab-TOAT training effect/benefit for the patient may transfer (or generalize) to either a new, previously untrained task, and/or a new environment like, for instance, the home environment, resulting in a behavioural change of using the affected arm in daily life. In regular arm-hand rehabilitation, but also during ReHab-TOAT, the therapist and the patient together look for possibilities to facilitate generalization of training progress towards other daily activities the patient may be able to perform when (s)he is at home. This includes performing the activities together and present tips to the patient on how (s)he can perform the activities more easily and also on how (s)he can perform the activities trained on (as well as similar activities) in his/her home environment. Patients’ positive experiences in task performance will boost their belief in arm-hand improvement, which may lead to: 1) integrating the affected hand in daily activities beyond clinical situations; and 2) support the transfer from trained tasks to other, often untrained, tasks [9, 37, 54]. Another important aspect is to create practice conditions to facilitate the patient in being less context-dependent, leading to improvements in adaptations in skill performance in new or different context conditions [45] like it is done in training phase 1, 4 and 6 of ReHab-TOAT. Furthermore, to even further promote patients’ self-efficacy, they get homework assignments after each session, thus continuing strength and mobility exercises and continuing using their affected arm in daily activities discussed. These homework assignments are also discussed with relatives of the patient, to include them and the home situation in the rehabilitation process and stimulate the use of the affected arm in daily life even more. The patient and the relatives may observe and encourage mastered skills. Prior to every ensuing training session, these new insights regarding the operational capabilities of the affected arm-hand are evaluated and discussed between the therapist and the patient.

***Appendix 4: Questionnaire for therapists*Questionnaire for therapists** **in ReHab-TOAT study**

You performed the same exercises / games that patients will perform after a stroke. We are very interested in your opinion about the content of the exercises / games. This questionnaire is divided into two parts. In the first part you are asked to rate the extent to which you agree with the statement. In the second part of the questionnaire you can formulate a short answer to the questions asked.

**Part 1**

Below you will find a number of statements about the training followed. Circle the number that you think best indicates how much you agree/disagree with the statement.

1. This arm-hand training was fun to do.

| 1 | 2 | | 3 | 4 | 5 | 6 | 7 |
| --- | --- | --- | --- | --- | --- | --- | --- |
| Strongly disagree | |  |  | Neutral |  |  | Strongly agree |

2. It was clear to me what to do during the exercises / games.

| 1 | 2 | | 3 | 4 | 5 | 6 | 7 |
| --- | --- | --- | --- | --- | --- | --- | --- |
| Strongly disagree | |  |  | Neutral |  |  | Strongly agree |

3. I think the exercises / games I had to do are also motivating for patients with a stroke.

| 1 | 2 | | 3 | 4 | 5 | 6 | 7 |
| --- | --- | --- | --- | --- | --- | --- | --- |
| Strongly disagree | |  |  | Neutral |  |  | Strongly agree |

4. I think the exercises / games I had to do are too complex for stroke patients.

| 1 | 2 | | 3 | 4 | 5 | 6 | 7 |
| --- | --- | --- | --- | --- | --- | --- | --- |
| Strongly disagree | |  |  | Neutral |  |  | Strongly agree |

5. I think the training duration is good for stroke patients.

| 1 | 2 | | 3 | 4 | 5 | 6 | 7 |
| --- | --- | --- | --- | --- | --- | --- | --- |
| Strongly disagree | |  |  | Neutral |  |  | Strongly agree |

6. I think the exercises / games I had to do are appropriate in intensity to the level of patients with a stroke.

| 1 | 2 | | 3 | 4 | 5 | 6 | 7 |
| --- | --- | --- | --- | --- | --- | --- | --- |
| Strongly disagree | |  |  | Neutral |  |  | Strongly agree |

7. I think this training for patients with stroke makes sense to do.

| 1 | 2 | | 3 | 4 | 5 | 6 | 7 |
| --- | --- | --- | --- | --- | --- | --- | --- |
| Strongly disagree | |  |  | Neutral |  |  | Strongly agree |

8. I found this arm-hand training boring to do.

| 1 | 2 | | 3 | 4 | 5 | 6 | 7 |
| --- | --- | --- | --- | --- | --- | --- | --- |
| Strongly disagree | |  |  | Neutral |  |  | Strongly agree |

9. I think that this training costs patients with a stroke too much energy.

| 1 | 2 | | 3 | 4 | 5 | 6 | 7 |
| --- | --- | --- | --- | --- | --- | --- | --- |
| Strongly disagree | |  |  | Neutral |  |  | Strongly agree |

10. I think this training is important for improving the dexterity of stroke patients.

| 1 | 2 | | 3 | 4 | 5 | 6 | 7 |
| --- | --- | --- | --- | --- | --- | --- | --- |
| Strongly disagree | |  |  | Neutral |  |  | Strongly agree |

11. I think that this training has added value in the arm-hand rehabilitation of patients with a stroke.

| 1 | 2 | | 3 | 4 | 5 | 6 | 7 |
| --- | --- | --- | --- | --- | --- | --- | --- |
| Strongly disagree | |  |  | Neutral |  |  | Strongly agree |

**Part 2**

Below you will find a number of open questions. We would like to ask you to answer these questions briefly.

**13. What did you think of the difficulty of the training? Which elements should be made easier / more difficult?**

**14. What did you think of the exercises / games?**

**15. Would you apply this training to patients with a stroke? Why yes, why not?**

**16. What do you like about this training?**

**17. What do you think could be improved about this training?**

**Thank you very much for your cooperation.**

## ***Appendix 5: Questionnaire for patients***

**Questionnaire for patients in ReHab-TOAT study**

We are very interested in your opinion about the exercises / games. This questionnaire is divided into two parts. In the first part you are asked to rate the extent to which you agree with the statement. In the second part of the questionnaire you can formulate a short answer to the questions asked.

**Part 1**

Below you will find a number of statements about the training followed. Circle the number that you think best indicates how much you agree/disagree with the statement.

1. This arm-hand training was fun to do.

| 1 | 2 | | 3 | 4 | 5 | 6 | 7 |
| --- | --- | --- | --- | --- | --- | --- | --- |
| Strongly disagree | |  |  | Neutral |  |  | Strongly agree |

2. I knew well what to do in the exercises / games.

| 1 | 2 | | 3 | 4 | 5 | 6 | 7 |
| --- | --- | --- | --- | --- | --- | --- | --- |
| Strongly disagree | |  |  | Neutral |  |  | Strongly agree |

3. The exercises/games encouraged me to do my best.

| 1 | 2 | | 3 | 4 | 5 | 6 | 7 |
| --- | --- | --- | --- | --- | --- | --- | --- |
| Strongly disagree | |  |  | Neutral |  |  | Strongly agree |

4. I found the exercises / games I had to do too difficult.

| 1 | 2 | | 3 | 4 | 5 | 6 | 7 |
| --- | --- | --- | --- | --- | --- | --- | --- |
| Strongly disagree | |  |  | Neutral |  |  | Strongly agree |

5. I was fine with the duration of the training.

| 1 | 2 | | 3 | 4 | 5 | 6 | 7 |
| --- | --- | --- | --- | --- | --- | --- | --- |
| Strongly disagree | |  |  | Neutral |  |  | Strongly agree |

6. The training was too hard for me.

| 1 | 2 | | 3 | 4 | 5 | 6 | 7 |
| --- | --- | --- | --- | --- | --- | --- | --- |
| Strongly disagree | |  |  | Neutral |  |  | Strongly agree |

7. I found this training useful to do.

| 1 | 2 | | 3 | 4 | 5 | 6 | 7 |
| --- | --- | --- | --- | --- | --- | --- | --- |
| Strongly disagree | |  |  | Neutral |  |  | Strongly agree |

8. I found this arm-hand training boring to do.

| 1 | 2 | | 3 | 4 | 5 | 6 | 7 |
| --- | --- | --- | --- | --- | --- | --- | --- |
| Strongly disagree | |  |  | Neutral |  |  | Strongly agree |

9. This training has cost me a lot of energy.

| 1 | 2 | | 3 | 4 | 5 | 6 | 7 |
| --- | --- | --- | --- | --- | --- | --- | --- |
| Strongly disagree | |  |  | Neutral |  |  | Strongly agree |

10. I think this training is important for improving my arm dexterity

| 1 | 2 | | 3 | 4 | 5 | 6 | 7 |
| --- | --- | --- | --- | --- | --- | --- | --- |
| Strongly disagree | |  |  | Neutral |  |  | Strongly agree |

11. I would like to do this again because it benefits me.

| 1 | 2 | | 3 | 4 | 5 | 6 | 7 |
| --- | --- | --- | --- | --- | --- | --- | --- |
| Strongly disagree | |  |  | Neutral |  |  | Strongly agree |

**Part 2**

Below you will find a number of open questions. We would like to ask you to answer these questions briefly.

**13. What did you think of the exercises / games?**

**14. What did you find easy about the training?**

**15. What did you find difficult about the training?**

**16. Would you like to do this training again? And can you indicate why (or not)?**

**17. What do you like about this training?**

**18. What do you think should be improved about this training?**

**Thank you very much for your cooperation.**
